# Supplementary material for: CKG: Improving ABSA with text augmentation using ChatGPT and knowledge-enhanced gated attention graph convolutional networks
Source: PLoS One. 2024 Jun 27;19(6):e0301508. doi: 10.1371/journal.pone.0301508 (PMC11210807; doi:10.1371/journal.pone.0301508)
Supplement: Attachment — Submitted filename: Response to Reviewers.docx [file pone.0301508.s001.docx]

Dear Editor,

Thank you for your letter and the reviewers’ comments concerning our manuscript. We sincerely appreciate receiving your letter and the reviewers' comments regarding our manuscript titled "Improving Aspect-Based Sentiment Analysis with Affective Knowledge Enhancements and Graph Adaptive Attention Mechanism"(PONE-D-23-24706R1). This research paper is now entitled “CKG: Improving Aspect-Level Sentiment Analysis with Text Augmentation using ChatGPT and Knowledge-Enhanced Gated Attention Graph Convolutional Networks”. Your valuable comments have been instrumental in guiding us towards the revision and improvement of our paper. We have thoroughly examined each comment and made the necessary revisions accordingly. We have reconstructed the article and virtually rewritten the entire paper. By the way, we have deleted the “reference.bib” file.

We would like to express our gratitude once again for your patience and guidance throughout this process.

Best regards,

Yapeng Gao, Lin Zhang, Yangshuyi Xu

Reviewer's Responses to Questions

Dear Reviewer #1,

Thank you for reviewing our paper and providing valuable feedback. We appreciate your constructive comments and suggestions. We have almost completely rewritten the entire article. We have carefully considered each point raised and have made the following revisions to address your concerns:

1. We have rewritten the abstract section of the original manuscript, summarizing the issues present in the field of aspect-level sentiment analysis from previous research and our corresponding solutions in the revised Abstract section.

2. We have reorganized the paper to provide a more balanced explanation of the proposed method, giving appropriate attention to the methods used.

3. We have systematically reviewed and addressed the improper word usage and grammar errors throughout the article. It is worth mentioning that we have almost completely rewritten the entire paper.

4. In section 4.4 "Results," we have included Table 5, which compares the performance of our model with some other models in the field of aspect-level sentiment analysis. This table provides a comprehensive comparison of our model's performance. In section 4.5 "Ablation Study," we have presented Table 6, which visually presents the results of our model's ablation experiments. Additionally, we have provided explanations regarding the configuration of model-related layers and encoding choices.

5. To enhance the comparison of the proposed method, we have incorporated more exciting methods and presented the results accordingly. In the section 4.4 "Results" of the paper, we have compared our model with traditional and recent mainstream models, using commonly accepted evaluation metrics in the field of aspect-level sentiment analysis. We have analyzed the comparative results and provided insights based on these evaluation standards, which have achieved a consensus in the field. In the section 4.7 "Attention Visualization" and the section 4.8 "Case Study", we have further elaborated on the effectiveness of our model using a combination of images and textual explanations. These sections provide visual and descriptive evidence to support the efficacy of our model.

6. We have revised the references and included more recent sources to ensure the relevance and up-to-dateness of the cited literature.

7. In section 5 "Conclusion", we have supplemented the future research directions.

Once again, we thank you for your valuable feedback, which has significantly improved the quality of our paper. We hope that the revisions adequately address your concerns and improve the overall clarity and presentation of our work.

Dear Reviewer #2,

Thank you for your thorough review of our paper. We appreciate your valuable feedback and have carefully considered each of your points. We have almost completely rewritten the entire article. We have made the following revisions to address your concerns:

1. Based on your feedback, we have restructured the Abstract section, highlighting the existing shortcomings in the past research and our research focus. We have ensured a clear and concise correspondence between them.

2. We apologize for such a basic mistake. We have rectified this error and revised the article.

3. We have conducted a detailed review of the image labels throughout the entire document, ensuring that each image is numbered clearly and accurately.

4. You are absolutely right, and I apologize for the mistake. We have meticulously derived all the formulas in the document, ensuring their accuracy. The corrected formulas can be found on line 246.

5. We have provided the sources for all the formulas that were not originally proposed by us.

6. We have adjusted the expression of this formula (Eq3) and reviewed other text-containing formulas to ensure their accuracy.

7. We have furnished the conclusion with concrete results to provide a more comprehensive summary of our study.

8. Firstly, in the Results section (4.4), we have provided a demonstration of the effectiveness of our experimental results. Secondly, in sections 4.5 to 4.8, we have discussed and analyzed our ablation experiments and the improvement achieved by our model. We have also included examples of practical applications.

9. The usage limit of ChatGPT can indeed affect our research efficiency. As mentioned in the paper, the "expert review of text generation quality" required communication and incurred a certain time cost. Additionally, even with specified evaluation metrics, human subjectivity can still influence the final judgment. Therefore, we are actively considering an effective and quantifiable evaluation method. In the Conclusion section (5), we elaborate on this matter.

10. In the Conclusion section of the paper, we have provided an exposition on the future research directions.

We appreciate your feedback, which has significantly helped improve the quality and clarity of our paper. We hope that the revisions adequately address your concerns and enhance the overall contribution of our work. Thank you once again for your valuable input.
