# Supplementary material for: CKG: Improving ABSA with text augmentation using ChatGPT and knowledge-enhanced gated attention graph convolutional networks
Source: PLoS One. 2024 Jun 27;19(6):e0301508. doi: 10.1371/journal.pone.0301508 (PMC11210807; doi:10.1371/journal.pone.0301508)
Supplement: Attachment — Submitted filename: Response to Reviewers_2.docx [file pone.0301508.s002.docx]

Dear Editor,

Thank you for your letter and the reviewers’ comments concerning our manuscript. We sincerely appreciate receiving your letter and the reviewers' comments regarding our manuscript titled " CKG: Improving ABSA with Text Augmentation using ChatGPT and Knowledge-Enhanced Gated Attention Graph Convolutional Networks" (PONE-D-23-24706R1). Your valuable comments have been instrumental in guiding us towards the revision and improvement of our paper. We have thoroughly examined each comment and made the necessary revisions accordingly.

We would like to express our gratitude once again for your patience and guidance throughout this process.

Best regards,

Yapeng Gao, Lin Zhang, Yangshuyi Xu

Reviewer's Responses to Questions

Dear Reviewer #1,

Thank you for your recognition of our research. We are confident that with your meticulous academic approach, you will undoubtedly be able to generate further achievements.

Dear Reviewer #2,

Thank you once again for your valuable feedback. Your meticulous attention to detail has deeply touched us. Based on your suggestions, we have made the following revisions:

1. We have included an explanation of the abbreviation ABSA in the abstract, and replaced the existing content throughout the entire manuscript while also verifying other abbreviations.

2. We have made modifications to the grammar and expressions in the manuscript, and have indicated these changes in the "Revised Manuscript with Track Changes."

3. We have indicated the source of the equations and acknowledge your reminder.

4. Thank you for your attention to detail. We have made the necessary correction to the erroneous expression, replacing "nd" with "and".

5. We have corrected the initial term "algorithm" to "Algorithm" and reviewed other similar usage for any potential errors.

6. At the corresponding locations, we have populated the empty spaces.

7. Based on your feedback, we have made adjustments to all the formulas by converting them into italics format.

Thank you for acknowledging our research work, which motivates us to pursue further investigations. With your guidance, we believe this paper can yield more remarkable outcomes and reach a wider scholarly audience. Wishing you continued success in your endeavors.

.
